# Paclitaxel biological synthesis promotes the innovation of anti‐cancer drugs

**DOI:** 10.1002/ctm2.70230

**Published:** 2025-02-09

**Authors:** Xiaolin Zhang, Gang Liu, Jianbin Yan

**Affiliations:** ^1^ Shenzhen Branch, Guangdong Laboratory of Lingnan Modern Agriculture, Key Laboratory of Synthetic Biology, Ministry of Agriculture and Rural Affairs, Agricultural Genomics Institute at Shenzhen Chinese Academy of Agricultural Sciences Shenzhen China; ^2^ School of Pharmaceutical Sciences Tsinghua University Beijing China

1

Paclitaxel, as a star natural drug with significant anti‐tumour activity, has become the frontline chemotherapy medication to treat various cancers since its advent in 1992.^1^ Different from other anticarcinogens, paclitaxel uniquely promotes the assembly of tubulin subunits into stable, non‐dynamic states that impede cancer cell proliferation, thereby effectively controlling the development of the disease. With the increasing number of cancer patients worldwide and the continuous advancement of medical technology, the market demand for paclitaxel is expected to expand continually. The market for the paclitaxel injection alone is estimated to reach 15.8 billion USD by 2032.^2^ Furthermore, integrating multiple disciplines, including medicine, biology and materials science, has the potential to expand the applications of paclitaxel significantly into medical devices. As an active pharmaceutical ingredient in drug‐coated balloons and stents, paclitaxel is crucial in inhibiting intimal proliferation and preventing in‐stent restenosis, thus providing a novel treatment option for patients with cardiovascular disease. This application improves the efficacy of paclitaxel and expands its clinical applications.

As precision medicine continues to advance, the personalisation of paclitaxel treatment is set to become a key focus for the future.^3^ Utilising advanced genomic and phenomic analysis techniques enables the precise identification of tumour types in patients. This capability allows for the development of tailored treatment plans, which can significantly enhance the therapeutic efficacy of the medication and improve the overall quality of life for patients. The ongoing optimisation of paclitaxel in combination with other medications and therapies will continue to advance, with an emphasis on enhancing treatment efficacy and targeting capability while minimising adverse effects. The advancement of paclitaxel precision medicine necessitates the enhancement of its yield and purity (Figure 1A). More crucially, it involves developing new paclitaxel derivatives that improve patient compliance with medication regimens.

**FIGURE 1:**
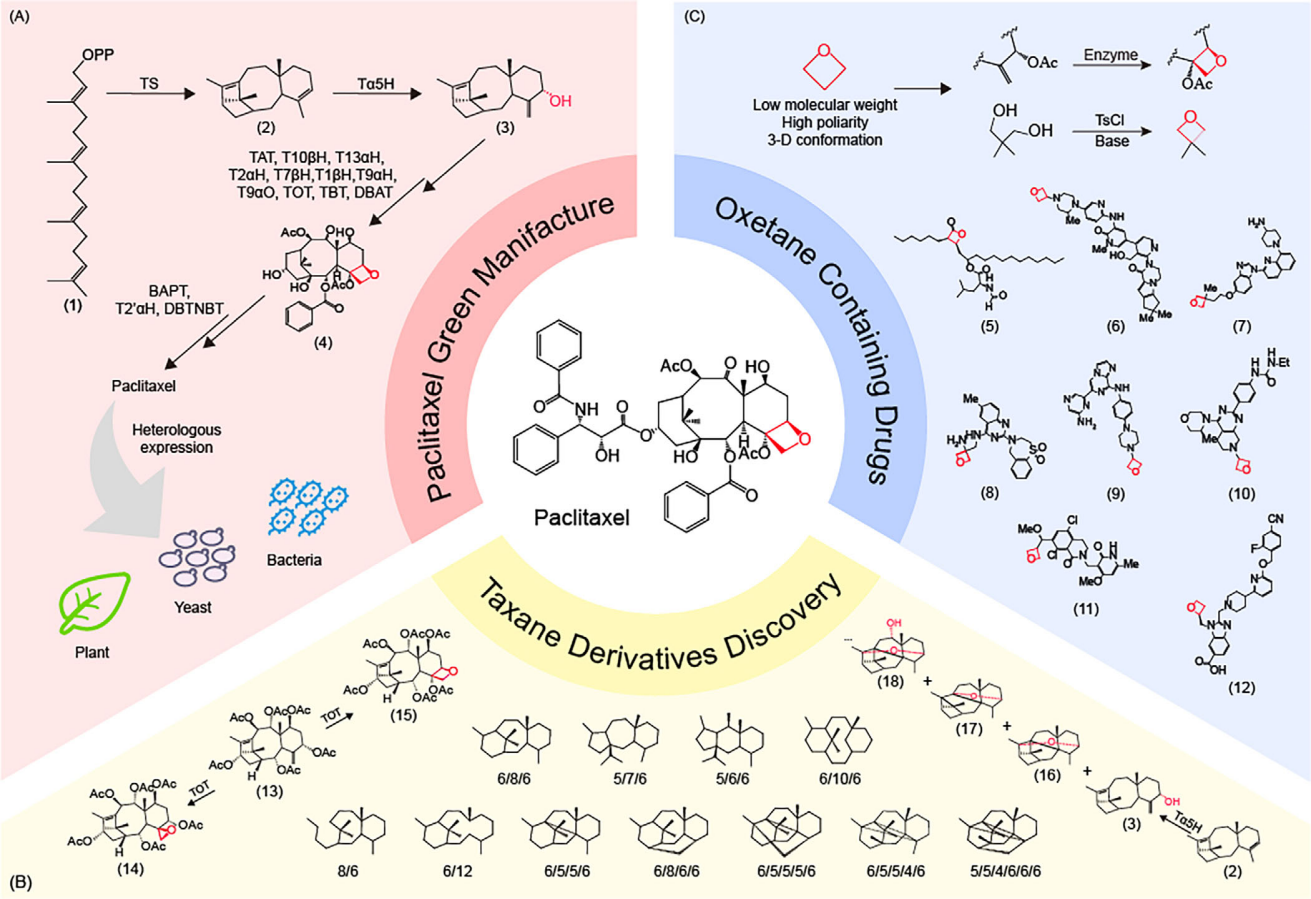
Development of paclitaxel‐related drugs from green manufacture to oxetane motif utilisation and taxane derivatives discovery. (A) Elucidation of biological synthesis pathway promotes sustainable paclitaxel production via heterologous expression in chassis such as plants, yeast and bacteria. (B) Catalytic versatility of enzymes, as exemplified by Taxane Oxetanase (TOT) and Taxadiene 5α‐hydroxylase (T5αH), and the substrate promiscuity, as demonstrated by diverse taxane ring systems where nomenclature reflects the number of carbons in the taxane skeleton rings (e.g., a 6/8/6 ring system indicates a skeleton composed of three rings with 6, 8 and 6 carbon atoms, respectively), jointly facilitate the discovery of novel taxane derivatives. (C) Oxetane motif plays a crucial role in creating novel drug candidates with diverse properties and potential therapeutic applications. (1) geranylgeranyl diphosphate, (2) taxadiene, (3) taxadiene‐5α‐ol, (4) baccatin III, (5) orlistat, (6) fenebrutinib, (7) crenolanib, (8) ziresovir, (9) lanraplenib, (10) GDC‐0349, (11) PF‐06821497, (12) danuglipron, (13) taxadiene hexa‐acetate, (14) baccatin I, (15) 1‐dehydroxybaccatin IV, (16) 5(12)‐oxa‐3(11)‐cyclotaxane and (17‒18) new di‐oxidised taxadiene varieties.

The rapid development of synthetic biology makes it possible to efficiently synthesise and directly generate novel paclitaxel derivatives tailored to different medical needs. It is reported that 600 taxoids have been identified from various *Taxus* species,^4^ among which about 24 taxoids exhibit cytotoxicity to tumour cells, thus providing a promising resource for discovering new agents with potent cytotoxic properties and reduced susceptibility to resistance. Recent breakthroughs in paclitaxel biosynthesis open new avenues for developing paclitaxel derivatives.^5^, ^6^ The enzymes that play a pivotal role in paclitaxel synthesis, especially those belonging to the cytochrome P450 family (CYP450s), demonstrate impressive catalytic versatility and remarkable substrate promiscuity.^7^ T5αH, hypothesised to catalyse the second step in the paclitaxel synthesis pathway, generates approximately 21 oxidised taxanes when expressed in *Saccharomyces cerevisiae*.^8^ Taxane Oxetanase 1 (TOT1), the enzyme responsible for the oxetane ring formation, was discovered to catalyse the formation of tricyclic products as well.^5^ The diverse catalytic products greatly enrich the pharmaceutical landscape to advance paclitaxel (Figure 1B).

The oxetane structure of paclitaxel plays a crucial role in enhancing its binding affinity to microtubules, consequently increasing its cytotoxic efficacy against tumour cells.^9^ In recent years, the oxetane motif has attracted considerable interest in synthetic chemistry, particularly its pharmaceutical applications, because the notable polarity and pronounced three‐dimensional conformation of oxetanes can enhance pharmacokinetic properties, leading to advantageous modifications in critical parameters such as pKa, LogD and solubility. In addition to taxoid drugs such as paclitaxel, docetaxel and cabazitaxel, orlistat is another FDA‐approved oxetane‐containing medication, noted for its anti‐obesity properties. The oxetane ring in orlistat helps maintain its molecular shape and enhances the drug's binding affinity to the active sites of pancreatic lipases. To date, seven oxetane‐containing drugs, including crenolanib, fenebrutinib, ziresovir, lanraplenib, danuglipron, GDC‐0349 and PF‐06821497, are under clinical trials, highlighting the versatility of the oxetane motif in medicinal chemistry.^10^ The oxetane scaffold has long been associated with significant synthetic challenges due to the limited availability of oxetane sources and the scarcity of efficient methods for their integration into medicinal chemistry. Remarkably, the discovery of TOT1,^5^ which enables the direct synthesis of oxetane rings from alkene precursors, presents a groundbreaking alternative for introducing oxetane motifs, offering a promising pathway for advancing research in this area. This advancement provides synthetic chemists with a more efficient and innovative method for integrating this valuable structural motif into pharmaceutical candidates (Figure 1C).

Over the past years, the application of paclitaxel has encountered limitations due to challenges related to its complex synthesis and associated manufacturing issues, impacting both availability and cost. However, recent advancements in understanding the biosynthetic pathways of paclitaxel present significant opportunities for developing sustainable and efficient bio‐manufacturing processes. Furthermore, the integration of molecular biology, biochemistry, systems biology and metabolic engineering presents an opportunity to develop a robust framework focused on optimising chassis customisation and fermentation processes. This comprehensive approach could certainly enhance the production of paclitaxel and its derivatives, thereby allowing paclitaxel to demonstrate its distinctive value and relevance across a broader array of applications, inspiring innovative taxane‐based therapeutics.

## AUTHOR CONTRIBUTIONS

Jianbin Yan conceived the review. Xiaolin Zhang drew the illustration and wrote the manuscript. Gang liu and Jianbin Yan revised the manuscript. All authors edited and approved the manuscript.

## CONFLICT OF INTEREST STATEMENT

The authors declare they have no conflicts of interest.
